# Chronic Ethanol Feeding Modulates Inflammatory Mediators, Activation of Nuclear Factor-****κ****B, and Responsiveness to Endotoxin in Murine Kupffer Cells and Circulating Leukocytes

**DOI:** 10.1155/2014/808695

**Published:** 2014-01-29

**Authors:** Miriam Maraslioglu, Elsie Oppermann, Carolin Blattner, Roxane Weber, Dirk Henrich, Christian Jobin, Elke Schleucher, Ingo Marzi, Mark Lehnert

**Affiliations:** ^1^Department of Trauma, Hand and Reconstructive Surgery, J.W. Goethe University, 60590 Frankfurt/Main, Germany; ^2^Department of Metabolic Physiology, J.W. Goethe University, 60439 Frankfurt/Main, Germany; ^3^Department of General and Visceral Surgery, J.W. Goethe University, 60590 Frankfurt/Main, Germany; ^4^Department of Medicine, Pharmacology and Immunology-Microbiology, University of North Carolina at Chapel Hill, Chapel Hill, NC 27599-7290, USA

## Abstract

Chronic ethanol abuse is known to increase susceptibility to infections after injury, in part, by modification of macrophage function. Several intracellular signalling mechanisms are involved in the initiation of inflammatory responses, including the nuclear factor-**κ**B (NF-**κ**B) pathway. In this study, we investigated the systemic and hepatic effect of chronic ethanol feeding on *in vivo* activation of NF-**κ**B in NF-**κ**B^EGFP^ reporter gene mice. Specifically, the study focused on Kupffer cell proinflammatory cytokines IL-6 and TNF-**α** and activation of NF-**κ**B after chronic ethanol feeding followed by *in vitro* stimulation with lipopolysaccharide (LPS). We found that chronic ethanol upregulated NF-**κ**B activation and increased hepatic and systemic proinflammatory cytokine levels. Similarly, LPS-stimulated IL-1**β** release from whole blood was significantly enhanced in ethanol-fed mice. However, LPS significantly increased IL-6 and TNF-**α** levels. These results demonstrate that chronic ethanol feeding can improve the responsiveness of macrophage LPS-stimulated IL-6 and TNF-**α** production and indicate that this effect may result from ethanol-induced alterations in intracellular signalling through NF-**κ**B. Furthermore, LPS and TNF-**α** stimulated the gene expression of different inflammatory mediators, in part, in a NF-**κ**B-dependent manner.

## 1. Introduction

Every fifth patient treated in hospital has a history of alcohol abuse [1], about 11 million people in the UK are estimated to regularly have an alcohol intoxication [2], and alcoholic liver disease (ALD) is an important outcome factor after trauma and elective surgery [3, 4]. Interestingly, chronic but not acute alcohol abuse adversely affects outcome at least in trauma patients [1, 5], and, besides, about 20% of alcoholics develop fibrosis and subsequent cirrhosis [6]. In contrast to the beneficial effect of moderate alcohol consumption, above all red wine, namely, the reported decrease of cardiovascular diseases [7, 8], these patients cope with complications such as high blood pressure, stroke, and an increased susceptibility to infections. In these patients it is widely accepted that bacteremia in blood is one of the key causes of liver injury.

Chronic ethanol abuse is known to cause disruption of the intestinal mucosal layer, leading to an increased permeability to gut-derived bacteria [9–13]. Once in the liver, endotoxin (LPS), a component of the wall of Gram-negative bacteria, binds to Toll-like receptor 4 (TLR4) and affects an intracellular signalling cascade resulting in NF-*κ*B activation, which in turn leads to release of hepatotoxic TNF-*α* [14, 15].

Another mechanism, by which liver damage is caused, is the activation of liver sessile Kupffer cells. A variety of subsequent reactions leading to cell injury exist, most notably for this study the generation and release of reactive oxygen species (ROS) and of pro-inflammatory mediators [13, 16–20]. The former occurs by catalytic activity of the transmembrane NADPH oxidase superoxideanion, which is an intermediate of ethanol metabolism, and the cytosolic NADPH oxidase [9, 21]. Kupffer cells do not only express various receptors for phagocytosis but, on activation, also produce multiple inflammatory mediators [e.g., interleukin-1*β* (IL-1*β*), IL-6, tumor necrosis factor-*α* (TNF-*α*)], mainly induced by TLR4 signaling. The TLR4 pathway downstream results in activation of transcription factors, such as nuclear factor-*κ*B (NF-*κ*B).

NF-*κ*B can act as an early transcription factor by modulation gene expression as no de novosynthesis is required. In most cells it is located in the cytoplasm as latent inactive I*κ*B-bound complex and as p50/p65 heterodimer [22]. NF-*κ*B-activating agents can induce the phosphorylation of I*κ*B inhibitory proteins, targeting them for rapid degradation through the ubiquitin-proteasome pathway and releasing NF-*κ*B to enter the nucleus where it modulates gene expression [23, 24].

In the present study we wanted to determine which role NF-*κ*B plays in ethanol-induced liver injury and furthermore in activated Kupffer cells after chronic ethanol feeding. Therefore we used a NF-*κ*B enhanced EGFP (enhanced green fluorescent protein) reporter gene mouse. As a second goal we experimentally tried to investigate the influence of ethanol preexposure (*in vivo*) on the reactivity of Kupffer cells to an *in vitro* LPS challenge.

## 2. Material and Methods

Male* cis-*NF-*κ*B^EGFP^ reporter gene mice and C57BL/6 mice were exposed to chronic EtOH intake. After the 4-weeks lasting pair-feeding regime, liver tissue samples were taken to measure steatosis, histopathological changes, NF-*κ*B activity, release of pro-inflammatory cytokines, and expression of inflammatory NF-*κ*B target genes. Blood samples were taken to measure systemic cytokines (IL-6, MCP-1, and TNF-*α*), AST (aspartate aminotransferase), and the expression of leukocyte surface markers (CD11b) and NF-*κ*B. In another experimental approach, liver was perfused for isolation of Kupffer cells from ethanol-fed (EtOH) and pair-fed mice, which were subsequently stimulated with LPS or TNF-*α*, respectively. Cytokines (IL-6, TNF-*α*), inflammatory NF-*κ*B target genes, and the receptor density of CD11b and CD68 (Scavenger) and NF-*κ*B in the Kupffer cell populations were measured.

### 2.1. Animals

Male *cis*-NF-*κ*B^EGFP^ mice (C57BL/6 background) were kindly provided by Christian Jobin, Chapel Hill, NC, USA, and bred in pathogen free conditions at Mfd Diagnostics (Wendelstein, Germany). In this gene targeted mouse strain, EGFP expression is under the transcriptional control of NF-*κ*B *cis*-elements; therefore NF-*κ*B binding results in transcription of EGFP [25]. At 6–8 weeks of age, weighing 20–25 g, they were delivered to our animal facility. Specific pathogen-free wild-type (WT) C57BL/6J mice (Janvier, Le Genest-Saint-Isle, France) served as controls. All animals were housed in separate individual, filter-top cages in an air flow, light (12 h light/12 h dark cycle), and temperature controlled room with free access to food and water. Animal protocols were approved by the Veterinary Department of the Regional Council in Darmstadt, Germany.

### 2.2. Experimental Model

Chronic ethanol feeding protocol: mice were acclimatized to our facility for 7 days after arrival, were randomly divided into pairs, and then assigned to a 4-week pair-feeding regime of standard Lieber-DeCarli diet (Ssniff Spezialdiäten; Soest, Germany) supplemented with either maltodextrin (control group) or ethanol 6.3% (vol/vol) (EtOH group) [26, 27]. Ethanol-fed mice were allowed free access to EtOH-supplemented diet. The amount of ingested diet was determined and an equal volume of maltodextrin-supplemented diet was supplied to the pair-fed animal. Accordingly, isocaloric feeding of each individual mouse was warranted. In selected experiments mice were fed standard laboratory chow, to control for the effects of the Lieber-DeCarli diet. As rodents naturally have an aversion against EtOH, the mice in this experiment were fed a liquid diet, with a gradual increase in the dose of EtOH starting with 1.75% (v/v) for 5 days, then increasing the dose to 2.63%, 3.5%, 4.38%, and finally 6.3% (v/v). This regimen reflects chronic ethanol abuse in humans, beginning with low volumes and increasing over time. Animal preparation: sacrifice and collection of tissue and blood samples: after 28 days of feeding Lieber DeCarli diet mice were weighed and anesthetized with isoflurane (Forane isoflurane, Abbott; Wiesbaden, Germany) under a continuous flow of 1.5 L/min by a mask. Laparotomy was carried out after sterilizing the abdomen and thorax with 70% EtOH by making a median incision 1 to 2 cm above the hind legs and continuing up to sternum, followed by a horizontal incision on each side ending at the rib cage. A 24-gauge needle was inserted in the IVC (inferior vena cava) and whole blood was withdrawn and collected. After disrupting portal vein, liver was perfused with Ringer's solution, excised, and weighed to determine the liver/body ratio. After removal of the gall bladder, a section of the liver's median lobe was embedded in Tissue-Tek O.C.T Compound (Sakura Finetek; Helsinki, Finland) for cryosections. Then the left lobe was infused and fixed with 4% buffered Zn-Formalin and subsequently embedded in paraffin, sectioned (7 *μ*m), and stained with hematoxylin-eosin (HE). The remaining liver lobes were cut into small pieces, snap-frozen in liquid nitrogen, and stored at −80°C for subsequent examination. In another experimental approach, liver was perfused with ice-cold Hank's Buffered Salt Solution (HBSS; Gibco; w/o Ca^2+^ and Mg^2+^) for 5 min for isolation of Kupffer cells from ethanol-, pair-, and chow-fed mice. After removing the gall bladder, liver was transferred to a sterile Petri dish containing HBSS (w/o Ca^2+^ and Mg^2+^) placed on ice until further preparation.

Groups: see Table 1.

### 2.3. Kupffer Cell Preparation and Culture

Isolation of NPC (nonparenchymal cells) from liver tissue: For Kupffer cell preparation the removed liver tissue was gently cut into small pieces, approximately 2 mm × 2 mm in the Petri dish on ice, and washed in a 50 mL conical tube in cold HBSS (w/o Ca^2+^ and Mg^2+^) until the supernatant was clear. After replacement in the Petri dish on ice, liver specimens were minced further finely using a scalpel and mashed with a plunger of a 10 mL syringe. Tissue was subdivided into 2 portions; each was transferred in a C tube (Miltenyi Biotec; Bergisch Gladbach, Germany) and subsequently resuspended in 15 mL Enzyme mix [50 mg/mL collagenase IV, 100U/mL DNaseI, 5 mM CaCl_2_, and 96 mL HBSS (w/o Ca^2+^ and Mg^2+^)]. Then, tissuse was dissociated gently by using Gentle MACS, program mouse liver (modified: “m_liver_01.02”, 73 s), followed by incubation at 37°C in a water bath with agitation mode set at the highest speed for 15 min. After repeating mechanical dissociation as described above followed by another incubation step for 15 min in water bath with agitation mode set at the highest speed, tissue was completely dissociated. Homogenizate was passed through a sterile nylon 70 *μ*m cell strainer (BD Biosciences; Heidelberg, Germany), washed twice with ice-cold DMEM [supplemented with 20% FCS, 50 *μ*g/mL gentamycin sulphate, 20 mM HEPES], pooled, and finally resuspended in 10 mL DMEM suppl. Cell suspensions were centrifuged at 17–21 ×g for 5 min to separate hepatocytes and the resulting supernatants from two mice per treatment group were pooled. Then they were layered carefully on a 50%/25% two-step Percoll gradient (GE Healthcare; Freiburg, Germany) in a 50 mL conical tube and centrifuged 15 min at 1800 ×g, 4°C (brake off) to separate nonparenchymal cells (NPCs) from parenchymal cells. Both interface fractions containing mainly Kupffer cells were transferred into DMEM suppl., washed twice, resuspended in 1 mL DMEM suppl. to determine cell number and viability by trypan blue exclusion, and found to be ~95%. Enrichment of F4/80^±^ Kupffer cells from NPC fraction: for selection of the F4/80 positive mononuclear cells fraction, cells suspensions were incubated with mouse FcR Blocking Reagent (Miltenyi Biotec) together with PE-conjugated anti-F4/80 Ab (Biolegend; San Diego, CA, USA) for 25 min at 4°C. After washing with MACS buffer [containing 2 mM EDTA, 0.5% BSA in PBS (w/o Ca^2+^ and Mg^2+^)], cells were magnetically labeled with anti-PE Microbeads (Miltenyi Biotec) for 15 min at 4°C and washed with MACS buffer. F4/80^+^ cells were separated over to sequential columns by positive selection using the MACS system (Miltenyi Biotec) according to manufacturer's recommendations. The eluate (F4/80^+^ cells), and the flow through (F4/80^−^ cells) were collected and purity of magnetic separation was determined by flow cytometric analysis on FACSCalibur (BD Biosciences; Heidelberg, Germany). Additionally vitality of isolated cells was evaluated by 7-AAD staining (BD Biosciences). Isolated Kupffer cells were suspended in DMEM suppl. and plated onto 24-well culture plates. After 2 h the media were replaced to remove nonadherent cells. After 16–18 h, cells were stimulated or not with either LPS (10 *μ*g/mL; Sigma; Deisenhofen, Germany) or TNF-*α* (500 ng/mL; R&D Systems), for 2, 4, or 24 h, respectively.

### 2.4. Measurement of Steatosis and Serum Enzyme Levels after Ethanol Feeding

Serum aspartate aminotransferase (AST) was detected using a dry chemistry analyzer (Spotchem EZ; Arkray, Philippines). Fat content was determined quantitatively by means of Soxhlet extraction technique as described elsewhere [28]. In brief, samples of dried and pulverized liver tissue were weighed and afterwards placed in an extraction thimble. Petroleum ether was used as solvent. By heating the water bath around the flask, the solvent is boiled and the vapour passes the condenser. Whereas ten reflux cycles were finished, whole fat has accumulated in the bottom flask and was weighed.

### 2.5. Analysis of Proinflammatory Changes due to Chronic Ethanol Intake

**Figure 1:**
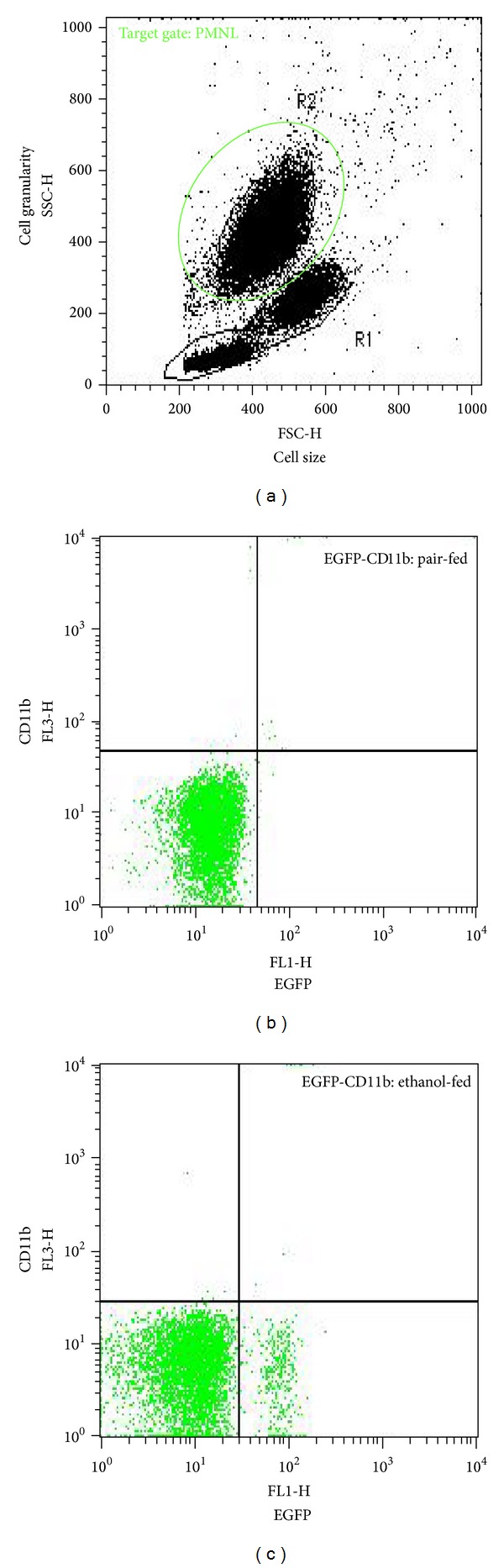
Gating strategy for the determination of PMNL. Representative FACS diagrams are shown. In (a), blood samples, obtained from *cis*-NF-*κ*B^EGFP^ after feeding Lieber-DeCarli diet for 4 weeks, were analyzed and the region (R2) was set according to the forward/side scatter characteristics (FSC/SSC) of PMNL. Dot plots depict EGFP expression versus isotype control CD11b staining from (b) pair- and (c) ethanol-fed mice.

Whole blood stimulation assay: monocyte activity was evaluated by whole blood stimulation assay with 10 *μ*g/mL endotoxin (LPS) from *Escherichia coli* 0127:B8 (Sigma) in RPMI 1640 medium (Sigma) and incubated for 24 h at 37°C and 5% CO_2_. A negative control lacking LPS for every assay was performed. Afterwards, blood cells were sedimented by centrifugation (2000 ×g, 10 min) and supernatants were collected and stored at −80°C. The IL-1*β* concentration was monitored using a Quantikine Mouse IL-1*β* ELISA kit following the manufacturer's instructions (R&D Systems). Quantification of cytokine levels: the release of IL-6, MCP-1, and TNF-*α* in plasma or culture supernatants was measured using flow cytometry with FACSCalibur (BD Biosciences; Heidelberg, Germany) and Mouse IL-6, MCP-1, and TNF-*α* Flex Set with a cytometric bead array according to the manufacturer's instructions (BD Biosciences). Concentrations of hepatic IL-6 in protein lysates extracted from snap-frozen liver tissue samples were determined using a Quantikine Mouse-IL-6 ELISA kit according to the manufacturer's instructions (R&D Systems). The ELISA 96-well microtiter plates were analyzed using a microplate reader Bio-Tek Ceres UV900C (Bio-Tek; Winooski, VT, USA). Determination of EGFP and CD11b cell surface expression in circulating neutrophils: Flow cytometry was performed to detect NF-*κ*B enhanced GFP and CD11b expression on the surface of leukocytes, as described in detail elsewhere [29]. Briefly, RBC-depleted peripheral blood cells were stained with anti-CD11b-PerCP-Cy5.5 (BD Biosciences). After washing with PBS containing 0.5% bovine serum albumin, cells were analyzed by a FACSCalibur (BD Biosciences). Polymorphonuclear neutrophils (PMNLs) were identified by their forward/side scatter characteristics (R2, Figure 1(a)). EGFP (FL-1) versus CD11b (FL-3) of the isotype control is presented (Figure 1(b): pair-fed; Figure 1(c): EtOH-fed). Data analysis was carried out using CellQuest Pro (BD Biosciences).

### 2.6. Visualization of *cis*-NF-*κ*B^EGFP^ Transcriptional Induction in Liver Tissue

EGFP in tissue specimens from *cis*-NF-*κ*B^EGFP^ mice was detected by epifluorescence microscopy. Tissue samples were fixed with 10% Zinc-Formalin for 24 h and paraffin-embedded. Sections were cut 5 *μ*m and EGFP expression was visualized by using the FITC reflector of Axio Observer Z1 (Carl Zeiss MicroImaging; Jena, Germany) with identical exposure times for each data point. Localization and cellular expression pattern of activated NF-*κ*B/GFP were further assessed by immunocytochemistry. Liver sections were fixed and cut as described and then incubated with anti-GFP antibody (1 : 400, 60 min, RT; Abcam; Cambridge, UK). An anti-rabbit horseradish peroxidase linked secondary antibody (30 min, RT; Histofine; Nichirei, Tokyo, Japan) and diaminobenzidine (Peroxidase EnVision Kit, DakoCytomation; Hamburg, Germany) were used to detect specific binding, followed by counterstaining with hematoxylin.

### 2.7. Detection of NF-*κ*B Activated Kupffer Cells

Paraffin-embedded liver sections (5 *μ*m) were deparaffinized and rehydrated. Macrophages were visualized using anti-F4/80-PE monoclonal antibody (Biolegend; San Diego, CA, USA) diluted 1 : 100 in phosphate-buffered saline (pH 7.4) containing 1% bovine serum albumin for 1 h. After washing with PBS, nuclei were counterstained with mounting medium containing 1.5 *μ*g/mL DAPI (Vector Laboratories; Burlingame, CA, USA). Fluorescence was visualized using multichannel fluorescence capturing with the reflectors DAPI-DNA (nuclei), FITC (EGFP), and Rhodamine (F4/80) of the Axio Observer Z1 microscope (Carl Zeiss MicroImaging; Jena, Germany). Representative images were captured from ten random fields with identical exposure times for each data point (×400).

### 2.8. Analysis of Kupffer Cell Subtypes by Staining of Characteristic Coreceptors

After isopycnic centrifugation with Percoll, a portion of the collected cell suspensions (~5 × 10^5^ cells) was stained with combinations of fluorochrome-conjugated antibodies against CD11b, CD68, and F4/80: F4/80-PE (Biolegend), CD11b-PerCP-Cy5.5 (BD Biosciences), and CD68-Alexa Fluor 684 (AbD Serotec). Fluorochrome-labeled isotype identical antibodies served as control. After 25 min of incubation at 4°C, cells were washed and percentage of Kupffer cell subtypes and amount of NF-*κ*B activation (EGFP, FITC channel) were determined using FACSCalibur flow cytometer (BD Biosciences).

### 2.9. Quantification of NF-*κ*B Activation in LPS Stimulated Kupffer Cells

To determine the proportion of NF-*κ*B activated Kupffer cells, 2 h, 4 h, or 24 h, respectively, after LPS stimulation, EGFP^+^ (green), F4/80^+^ (red), and colabeled (orange) cells were counted. Fluorescence was visualized using multichannel fluorescence. Images were taken with the reflectors FITC (EGFP) and Rhodamine (F4/80) of Axio Observer Z1 (Carl Zeiss MicroImaging). Representative images were captured from ten random fields with identical exposure times for each data point (×400).

### 2.10. Investigation on Gene Expression of Inflammatory NF-*κ*B Target Genes in Kupffer Cells of Ethanol-Fed Mice after LPS and TNF-*α* Challenge

To examine the expression of TNF-*α*, IL-6, matrix metalloproteinase-9 (MMP-9), CXCL-1, and NOS2, total RNA was extracted using the RNeasy-system (Qiagen; Hilden, Germany) according to the manufacturer's instructions, after collecting the supernatants from the LPS stimulated Kupffer cells. The residual amounts of DNA remaining were removed using the RNase-Free DNase Set according to the manufacturer's instructions (Qiagen). Quality and amount of the RNA were determined photometrically using the NanoVue Plus device (GE Healthcare; Munich, Germany). Reverse transcription was carried out subsequently with Omniscript (Qiagen; Hilden, Germany) using the AffinityScript PCR cDNA Synthesis Kit (Stratagene; La Jolla, CA, USA). qRT PCR reactions were performed using Stratagene MX3005p QPCR system (Stratagene) with specific primers for target genes (Table 2) and 18S ribosomal RNA as a reference gene, all purchased from SA Bioscience (SuperArray; Frederick, MD, USA). PCR reaction mixtures (25 *μ*L) were performed using 1X RT^2^ SYBR Green/Rox qPCR Master mix (SA Bioscience) according to manufacturer's instructions. Amplification of cDNA was initiated with 10 min of denaturation at 95°C followed by 40 cycles with 15 s denaturation at 95°C and 60 s annealing/extension at 60°C. A melting-curve analysis was applied to control the specificity of amplification products. Relative expression of each target gene's mRNA level was then calculated using the comparative threshold-cycle (CT) method (2^−ΔΔCT^ method). In brief, the amount of target mRNA in each sample was first normalized to the amount of *18S ribosomal* mRNA to give ΔCT and then to a calibrator consisting of samples obtained from the stimulation-Ctrl group. The relative mRNA expression of target genes is presented as fold increase calculated in relation to stimulation control (medium) after normalization to *18S ribosomal RNA.*

### 2.11. Statistical Analysis

Data are presented as mean ± SEM (standard error of the mean). A *P* value of less than 0.05 was considered significant. Differences between means were determined by one-way analysis of variance (ANOVA) followed by the Student-Newman-Keuls test as a post hoc test for multiple comparisons.

## 3. Results

### 3.1. Liver Injury due to Ethanol Feeding

**Figure 2:**
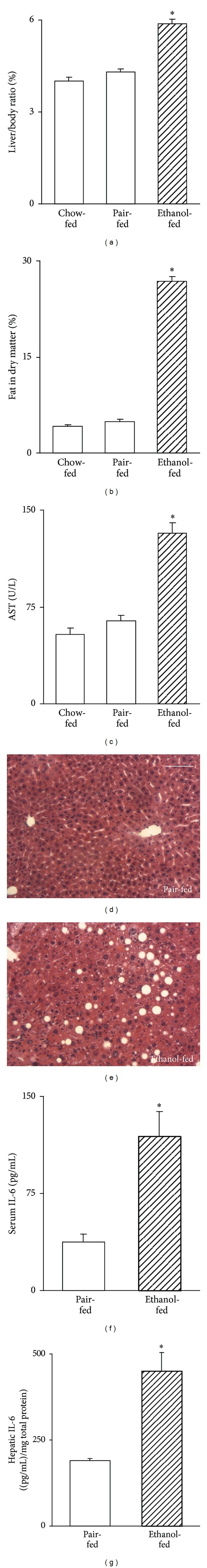
EtOH-containing liquid diet affects fatty liver and increased proinflammatory IL-6 release 4 weeks after feeding mice an ethanol (EtOH-fed) or control (pair-fed) Lieber DeCarli diet; blood samples and livers were harvested as described in Section 2. Chow-fed animals served as internal controls for the pair feeding approach. Data are given as mean ± SEM. *P* < 0.05 versus all. In (a), liver body ratio from pair-fed mice is presented. Hepatic dry fat content was quantified by means of Soxhlet technique as described in Section 2. section (b). Serum aspartate aminotransferase levels were measured (c). Representative photomicrographs of HE stained liver sections from (d) pair- and (e) ethanol-fed mice are presented. Bar equals 100 *μ*m. In (f), systemic levels of IL-6, and in (g), hepatic IL-6 proteins are shown. *P < 0.05 versus pair-fed Ctrl.

Feeding of ethanol containing Lieber DeCarli diet for 28 days increased the relative liver weight (liver/body weight ratio 4.3 ± 0.1 versus 5.85 ± 0.2, *P* < 0.05; Figure 2(a)). Quantification of dry fat content of whole liver tissue revealed an increase when compared to the control group (*P* < 0.05, Figure 2(b)). EtOH feeding caused an elevation in serum AST to 132.2 ± 8.1 U/L when compared to pair-fed mice (*P* < 0.05, Figure 2(c)). Feeding the maltodextrin containing Lieber-DeCarli diet revealed the same effect on the aforementioned physiological liver markers as feeding a regular chow food diet. These results demonstrate the effectiveness of the pair feeding approach to study the effects of ethanol feeding while an equicaloric condition is maintained and no hepatic changes are induced by the maltodextrin containing diet.

### 3.2. Effects of Ethanol Diet on the Local and Systemic Inflammatory Response

Chronic EtOH feeding caused a systemic inflammatory response, as determined by circulating levels of IL-6, MCP-1, and TNF-*α*. The concentration of IL-6 rose markedly in the EtOH-fed group when compared to the control group (119.14 ± 19.4 versus 37.73 ± 6.1 pg/mL, respectively, *P* < 0.05; Figure 2(f)). The same effect was observed for levels of MCP-1 (287.2 ± 45.4 versus 85.75 ± 28.7 pg/mL, *P* < 0.05; data not shown) as well as TNF-*α* (8.26 ± 2.2 versus 2.72 ± 1 pg/mL, *P* < 0.05; data not shown). Interestingly, chronic EtOH intake caused a local hepatic IL-6 release when compared to pair-fed mice with EtOH-free diet (450.87 ± 52.8 versus 189.39 ± 7.5 (pg/mL)/mg protein, *P* < 0.05; Figure 2(g)) but without histopathological evidence for steatohepatitis (Figure 2(e)).

LPS-stimulated monocyte cytokine production: the *in vitro* production of IL-1*β* in whole blood was higher in ethanol-fed *cis*-NF-*κ*B^EGFP^ mice when compared to pair-fed controls after LPS stimulation which was comparable to mice fed a regular chow diet (84.43 ± 31.4 versus 18.5 ± 3.3 pg/mL; *P* < 0.05; data not shown).

**Figure 3:**
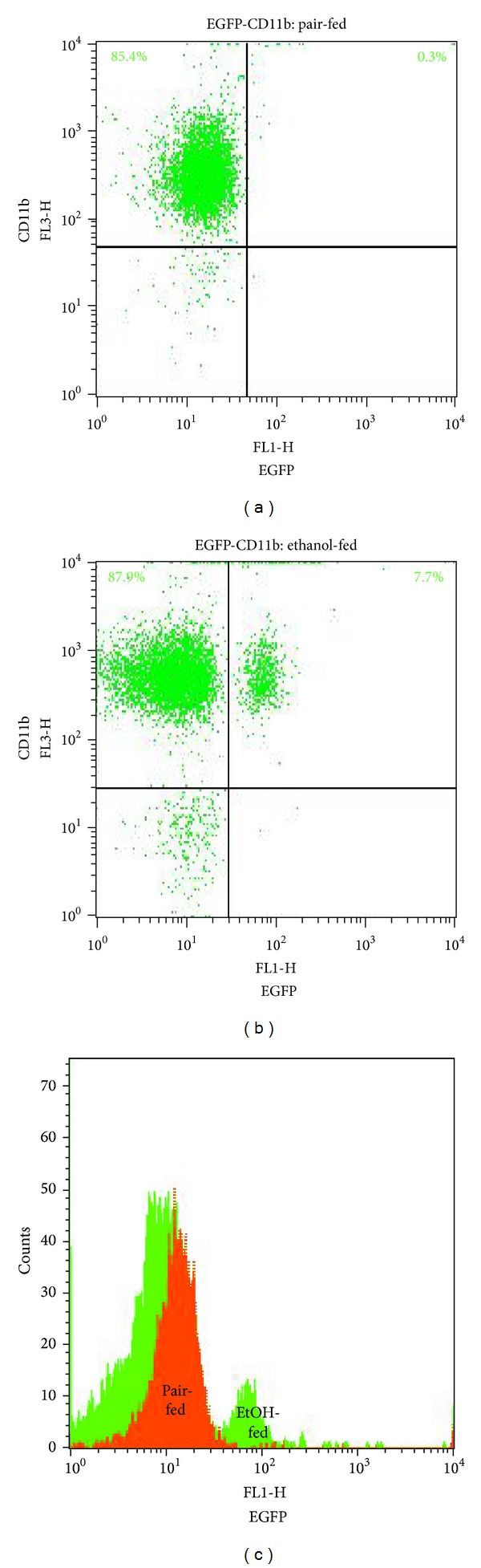
Surface expression of CD11b and transcriptional induction of the *cis*-NF-kB^EGFP^ transgene after chronic ethanol exposure. Representative FACS diagrams are shown. EGFP expressing versus CD11b^+^ PMNL, gated by their FSC/SSC properties as shown in Figure 1, were identified in a FL-1-FL-3 scattergram. The percentage of the indicated populations was determined through quadrant analysis of (a) pair- and (b) ethanol-fed mice. In (c), an overlay of FACS histograms, representative of five independent experiments, demonstrates elevated EGFP expression in PMNLs from ethanol pretreated mice. The orange-filled histogram depicts pair-fed controls and the green-filled graph ethanol-fed mice.

Ethanol feeding primed peripheral blood neutrophils: chronic EtOH intake activates circulating polymorphonuclear leukocytes (PMNLs) as indicated by FACS analysis. The expression of a prerequisite surface marker to migrate through the endothelium, the integrin Mac-1 (CD11b/CD18), and the expression of EGFP, representing NF-*κ*B activation, were investigated in peripheral blood samples collected after chronic ethanol feeding. After pair feeding, only 0.3% of PMNLs showed coexpression of CD11b/CD18 and EGFP and this part rose markedly to 7.7% after ethanol pretreatment. Total EGFP expression was increased in ethanol-primed CD11b^+^ PMNL (Figure 3(c)).

### 3.3. Intensified Expression of *cis*-NF-*κ*B^EGFP^ in Liver Tissue

**Figure 4:**
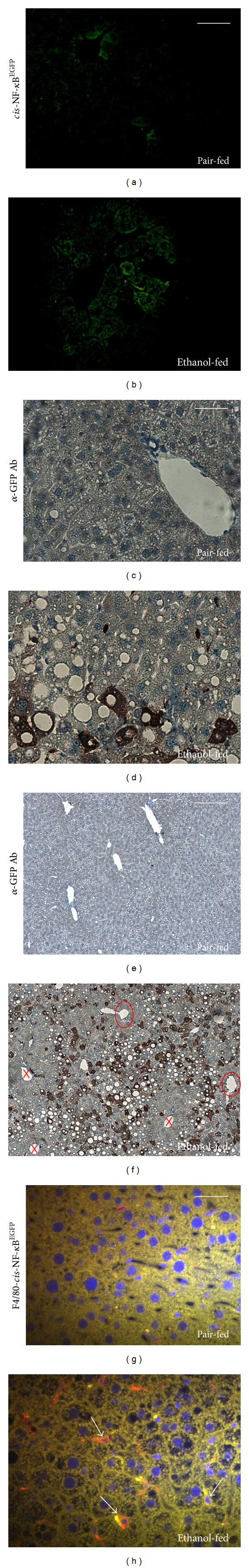
Effect of chronic ethanol on hepatic topography of * cis*-NF-*κ*B^EGFP^ transcriptional induction and hepatic macrophage activation of NF-*κ*B dependent EGFP expression was analyzed using fluorescence microscopy in livers harvested from *cis*-NF-*κ*B^EGFP^ mice and prepared as described in Section 2. Representative liver lobes from pair-fed ((a), (c), (e), and (g)) and ethanol-fed ((b), (d), (e), and (h)) mice are shown. Green fluorescence by EGFP represents NF-*κ*B activity ((a), (b): bar equals 100 *μ*m). Additional GFP antibody staining identifies the topography of NF-*κ*B activation ((c), (d): bar equals 50 *μ*m; (e), (f): bar equals 200 *μ*m). The encircled areas mark central veins; the crosses mark portal fields. Immunostaining for F4/80 (red fluorescence) identifies Kupffer cells in *cis*-NF-*κ*B^EGFP^ mice livers; overlay images show colabeled cells, marked by arrows ((g), (h): bar equals 20 *μ*m).

To assess the time and site specific expression of EGFP representing sites of NF-*κ*B activation after chronic ethanol abuse, paraffin-embedded liver sections were analyzed by epifluorescence microscopy. An increased NF-*κ*B transcriptional activity was present in EtOH treated mice (Figure 4(b)) compared to pair-fed mice (Figure 4(a)). Bias from hepatic autofluorescence was eliminated by immunostaining liver sections with an anti-GFP antibody and again more GFP was present after ethanol feeding (Figures 4(c)–4(f)). Furthermore, tissue was immunostained with F4/80 to identify mouse macrophages, mostly liver sessile Kupffer cells. After pair feeding, only F4/80 positive Kupffer cells were detected whereas, after ethanol feeding, the proportion of cells coexpressing EGFP and F4/80^−^ resulting in a yellow type fluorescence was largely elevated (Figures 4(g) and 4(h)). Interestingly, in ethanol-fed mice, EGFP-positive Kupffer cells were mainly located in periportal and midzonal areas, whereas EGFP-positive hepatocytes could be found mostly in pericentral and midzonal areas. These observations indicate that the ethanol-containing diet influences both quantity and topography of NF-*κ*B activation in hepatocytes and macrophages.

### 3.4. Expression of CD11b and CD68 on F4/80^+^ Kupffer Cells

Freshly isolated Kupffer cells demonstrated a significant surface expression of CD11b and CD68 with expression of all receptors more pronounced in macrophages from ethanol-fed mice (Table 3).

### 3.5. NF-*κ*B in Kupffer Cells Is Activated by Both Ethanol Feeding and *In Vitro* LPS Stimulation

**Figure 5:**
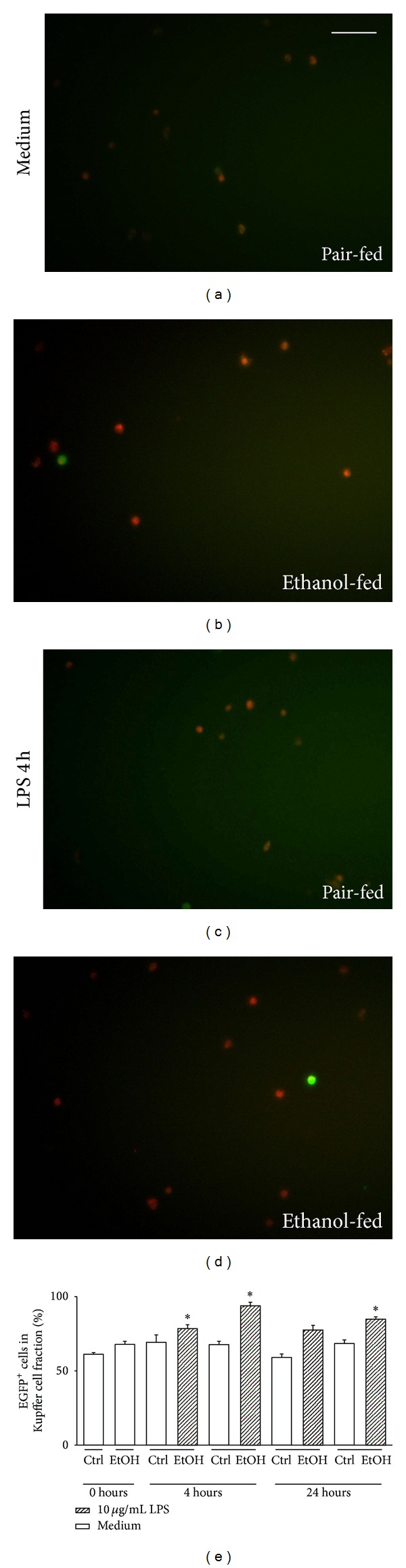
*cis*-NF-*κ*B^EGFP^ transcriptional induction in F4/80^+^ hepatic macrophages at 4, 24 h after *in vitro* LPS challenge prior to chronic ethanol feeding. Livers of *cis*-NF-*κ*B^EGFP^ mice were harvested 4 weeks after Lieber-DeCarli diet treatment and Kupffer cells were purified and cultured as described in Section 2. Cells were then stimulated with 10 *μ*g/mL LPS over a period of 24 hours. Red fluorescence (F4/80) labels Kupffer cells and green fluorescence of EGFP identifies cells expressing NF-*κ*B transcriptional activity at 4, 24 h after LPS stimulation. (a) and (b) show representative unstimulated controls whereas in (c) and (d) representative overlay images at 4 h after LPS stimulation are given (magnification, ×200). Colabeled (yellow) cells and F4/80 positive (red) cells were counted. The percentage of F4/80^+^EGFP^+^ cells in total F4/80^+^ cells is depicted in (e) at 4, and 24 h after LPS stimulation. Data shown are representative of five to eight separate experiments and are presented as mean ± SEM. *P < 0.05 versus Ctrl.

NF-*κ*B activation in isolated KC was enhanced after chronic ethanol feeding when compared to pair-fed mice (unstimulated Ctrl: Figure 5(a), unstimulated EtOH: Figure 5(b)). Both after pair feeding and ethanol diet, the proportion of activated KC was largely enhanced at 4 h after LPS stimulation (Ctrl: Figure 5(c), EtOH: Figure 5(d)). Interestingly, the percentage of KC with activated NF-*κ*B does not further increase at 24 h after LPS stimulation (Figure 5(e)).

### 3.6. NF-*κ*B Activation in Kupffer Cells Is Associated with Release of Proinflammatory Cytokines

**Figure 6:**
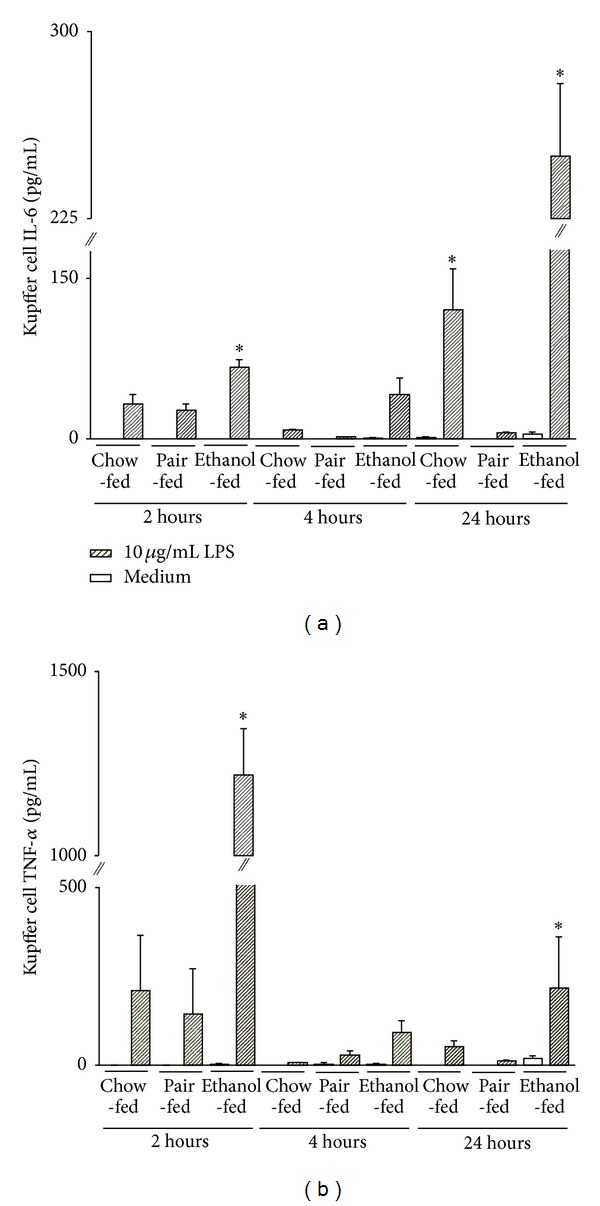
Proinflammatory cytokine production of F4/80^−^ hepatic macrophages following ethanol feeding in response to LPS or TNF-*α*. Kupffer cells were isolated from *cis*-NF-*κ*B^EGFP^ mice after 4 weeks of pair feeding regime, as described in Section 2, and stimulated with 10 *μ*g/mL LPS or 500 ng/mL TNF-*α*. Culture supernatants were collected at 2 h, 4 h, and 24 h after stimulation. IL-6 (a) and TNF-*α* (b) were measured by cytometric bead array (CBA). Data (mean ± SEM) are representative of five to eight independent experiments. *P < 0.05 versus Ctrl.

To analyze the inflammatory potential of Kupffer cells obtained from ethanol-fed mice and pair- and chow-fed controls, we measured the concentrations of several cytokines in the supernatants 2 h, 4 h, and 24 h after endotoxin stimulation. IL-6 release was the highest after 24 h LPS and EtOH diet (Figure 6(a)); TNF-*α* rose to the highest levels 2 h after LPS treatment and EtOH feeding (Figure 6(b)). Again, cytokine production of Kupffer cells from mice fed with the maltodextrin augmented Lieber DeCarli diet did not differ from mice fed with a regular chow diet. These results indicate that the increased NF-*κ*B activation in KC is associated with an increased production of inflammatory cytokines and, that the pair feeding of Lieber-DeCarli diet is a valuable tool to analyze even subtle changes in individual cell subsets with results that can be transferred to animals fed with the standard chow food. No significant differences were observed in both control groups.

### 3.7. LPS- and Ethanol-Induced Alterations in Expression of NF-*κ*B Controlled and Proinflammatory Genes

**Figure 7:**
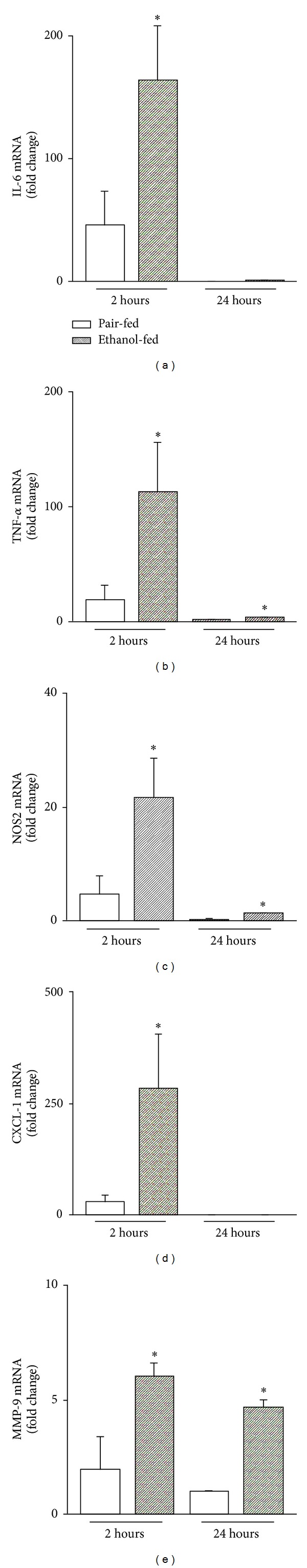
NF-*κ*B target gene expression in isolated Kupffer cells in response to LPS after ethanol feeding F4/80^+^ macrophages were isolated from livers of ethanol-, pair-fed mice, respectively, and stimulated with LPS (10 *μ*g/mL, Figure 7) for 2 h and 24 h. Supernatants were measured for mRNA levels of IL-6 (a), TNF-*α* (b), NOS2 (c), CXCL-1 (d), and MMP-9 (e) by RT-PCR. Data (mean ± SEM) are expressed as fold change compared with corresponding medium controls (unstimulated) and are representative of five to eight separate experiments. *P < 0.05 versus pair-fed control.

**Figure 8:**
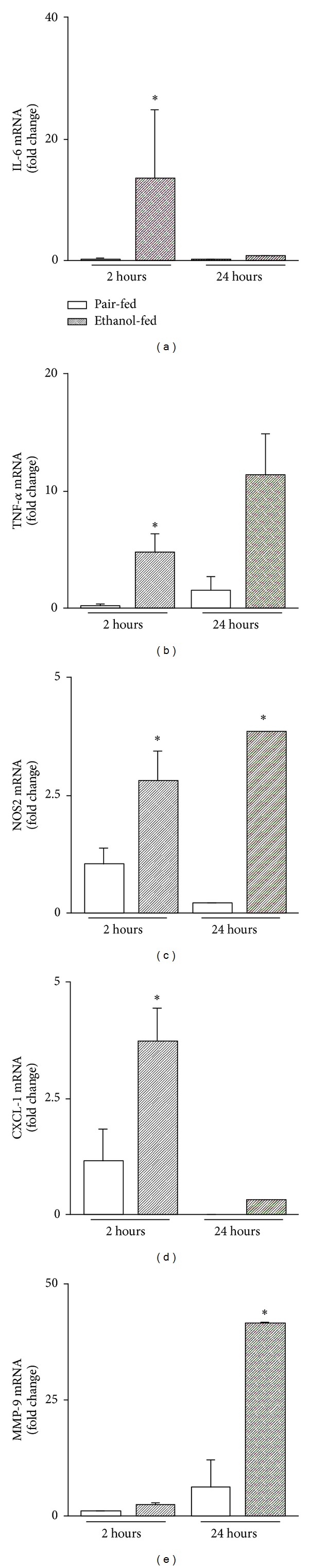
NF-*κ*B target gene expression in isolated Kupffer cells in response to TNF-*α* after ethanol feeding F4/80^+^ macrophages were isolated from livers of ethanol-, pair-fed mice, respectively, and stimulated with TNF-*α* (500 ng/mL) for 2 h and 24 h. Supernatants were measured for mRNA levels of IL-6 (a), TNF-*α* (b), NOS2 (c), CXCL-1 (d), and MMP-9 (e) by RT-PCR. Data (mean ± SEM) are expressed as fold change compared with corresponding medium controls (unstimulated) and are representative of five to eight separate experiments. *P < 0.05 versus pair-fed control.

LPS stimulation of macrophages is known to induce among others TNF-*α* mRNA expression through activation of the canonical NF-*κ*B pathway [22]. To investigate whether expression of TNF-*α* and of other NF-*κ*B related genes is increased in ethanol-primed Kupffer cells, gene expression was assessed by RT PCR. Nearly all analyzed genes, either after LPS stimulation (Figure 7) or after TNF-*α* stimulation (Figure 8), showed higher expressions after ethanol feeding alone and in combination with stimuli. Interestingly, stimulation with LPS and TNF-*α* resulted in a different activation pattern. 2 h after LPS stimulation the production of IL6 and TNF-*α* was largely elevated when compared to pair-fed controls (IL-6 mRNA: 164.1 ± 45.1 versus 46.0 ± 27.6, TNF mRNA: 113.0 ± 43.2 versus 18.9 ± 13.7, *P* > 0.05; Figures 7(a) and 7(b)).

## 4. Discussion

NF-*κ*B plays an integral role in liver injury and inflammation as the main consequence of acute and chronic ethanol consumption [12, 16, 18, 19, 30–33]. Our study demonstrates that the inflammatory response following chronic ethanol abuse is characterized by the activation of hepatic macrophages (Kupffer cells, Figures 4–8), monocytes (data not shown), and PMNL (Figure 3) and the upregulation of pro-inflammatory mediator synthesis (Figures 2 and 6). Using a transgenic NF-*κ*B^EGFP^ mouse model we present a strong association of these observations to the activation of NF-*κ*B *in vivo* (Figures 3–5). Furthermore, the percentage of Kupffer cells (KC) with activated NF-*κ*B does increase after LPS stimulation *in vitro* in ethanol-fed mice, indicating that chronic ethanol feeding does at least partially improve the ability of KC to react to a secondary stimulus such as incubation with LPS (Figure 5).

To investigate the hepatotoxic effect of chronic ethanol abuse, we used a voluntary, EtOH diet-feeding model, first described by Lieber and DeCarli, in 1967. This ad libitum model causes signs of steatosis and mild steatohepatitis [10, 34–38] that closely simulates that seen in humans following chronic ethanol consumption [39]. The final dose of 6.3% (v/v) in our model corresponds to 35% of the calorie intake as carbohydrates [35, 36]. Advantages compared to the intragastric gavage (IG) model are defined by better simulation of chronic EtOH consumption after voluntary feeding, avoidance of repeated surgery or stomach intubation, and preservation of a continuous metabolic rate in rodents [40]. Accordingly in our study, chronic EtOH intake led to a fatty liver, with increased liver to body weight ratios in ethanol-fed mice (Figure 2). Fatty liver also is the hallmark of ethanol induced liver injury in humans with EtOH metabolism in hepatocytes causing hyperplasia by the accumulation of free fatty acids in cytosol and in interstitial space (Figures 2(a) and 2(e)). Liver fat content (Figure 2(b)) was comparable to studies by others in C57BL/6 mice [10, 35]. HE staining of liver parenchyma (Figure 2(e)) as well as serum transaminase release (Figure 2(c)) revealed marked signs of steatosis and hepatocellular damage.

An increased systemic release of proinflammatory mediators such as IL-6, TNF- and macrophage chemoattractant protein- (MCP-) 1, activation of adhesion molecules such as CD11b in circulating neutrophils an increased hepatic IL-6 level was present in ethanol-fed animals, clearly reflecting steatohepatitis; however histomorphological sequelae were not present consistent with previous reports using the Lieber DeCarli-ethanol diet (Figure 2(e) versus Figure 2(g)) [34, 37]. Further, the amount of activated circulating leukocytes after ethanol feeding that coexpressed EGFP and the binding receptor CD11b/CD18 (Mac1) was elevated (Figure 3). Interestingly, these pathophysiological changes were also associated with an enhanced EGFP expression reflecting NF-*κ*B activation in ethanol-fed mice livers (Figures 4(b), 4(d), 4(f), and 4(h)). Hence, NF-*κ*B activation after ethanol pretreatment is also present in circulating neutrophils and seems to correlate with the local and systemic synthesis of NF-*κ*B-dependent mediators.

Our results also demonstrate a NF-*κ*B-dependent priming effect of an ethanol diet on F4/80 positive macrophages in the liver (Kupffer cells); incubation with both LPS and TNF-*α* resulted in a largely exaggerated release of pro-inflammatory mediators and expression of NF-*κ*B-dependent target genes (Figures 6–8). However, differences in the amount of NF-*κ*B positive KC are only seen at 4 h after LPS stimulation (Figure 5(e)). Accordingly, this study presents evidence that Kupffer cells are an important player in initiating an overwhelming pro-inflammatory immune reaction after chronic ethanol feeding.

Although NF-*κ*B is broadly accepted as a crucial factor in the regulation of the intracellular mechanisms after chronic ethanol abuse, there is, to our knowledge, no study that directly visualizes NF-*κ*B spatial and temporal activation pattern in the liver and in circulating and hepatic immune cells. However, the function and involvement of NF-*κ*B in different liver cell populations might be quite different. Using the NF-*κ*B^EGFP^ transgenic mice, we visualized the spatial NF-*κ*B activation in hepatic nonparenchymal cells and this differed profoundly compared to the topographical activation of NF-*κ*B in hepatocytes. EGFP expressing hepatocytes were typically found in the midzonal and pericentral regions in livers of ethanol-fed mice (Figures 4(b), 4(d), and 4(f)). Hepatocytes in the pericentral zones of the liver lobe are involved in ethanol metabolism. In contrast, hepatic Kupffer cells showed a strong NF-*κ*B activity in periportal and midzonal regions of the liver after chronic ethanol intake (Figure 4(h)) where they form the first line of defense against bacterial pathogen-associated molecular patterns (PAMPs), entering the portal circuit via the gut-liver axis, and endogenous damage-associated molecular patterns (DAMPs) generated at sites of sterile inflammation [41–44].

LPS stimulation of Kupffer cells increases TNF-*α* release and results in hepatocyte cell death and increased local synthesis of pro-inflammatory mediators, such as IL-6, IL-1, and TNF-*α* [13, 20, 45–47]. In our study, IL-6 release from ethanol pretreated Kupffer cells peaks at 24 h after KC stimulation, whereas peak of TNF-*α* release occurs already at 2 h after stimulation of ethanol pretreated KC (Figure 6). In parallel, detection of early inflammatory changes in isolated KC (mRNA expression: Figures 7(a)–7(e)) is also elevated after ethanol pretreatment. These differences in cytokine expression and production may be due, in part, to activation of NF-*κ*B in Kupffer cells since after 4 h of LPS stimulation a larger percentage of EGFP positive Kupffer cells in ethanol-fed mice are present, an effect that is attenuated at 24 h after LPS stimulation (Figure 5(e)). Therefore, ethanol pretreatment of KC affects cytokine expression and production profiles when compared to control fed animals and this effect may be partly due to activation of NF-*κ*B in Kupffer cells. Interestingly, acute ethanol intoxication exerts anti-inflammatory effects in the setting of resuscitated blood loss [19, 31, 48, 49]. Further studies are certainly needed to more specifically dissect the contribution of various cell types to the modulation of inflammatory responses after ethanol exposure.

Alcohol abuse plays a particular role in patients admitted to emergency services, for example, suffering from traumatic injury or massive bleeding, or after surgical interventions. The outcome and the incidence for multiple organ failure (MOF) or sepsis in acute ethanol intoxicated individuals differ from those of patients with a history of chronic ethanol abuse, mostly as a result of impaired host response [1, 16, 50–53]. Thus, the 24 h survival after trauma and the in-hospital mortality were worse in chronic ethanol abusers, and the percentage of individuals, suffering from a multiple organ failure (MOF), was 2-fold higher in victims with a cirrhotic liver when compared to acutely intoxicated patients [1]. A better outcome after binge-like ethanol consumption might be due to the activation status of innate immune cells. However, chronic ethanol affects a stimulation of the responsiveness of PMNL after a second challenge and therefore leads to an overwhelming multifactorial immune response [19].

## 5. Conclusions

Taken together NF-*κ*B activation in Kupffer cells seems to be of critical importance in the response of the innate immune system after chronic ethanol feeding. There is accumulating evidence that ethanol-primed macrophages show an altered cytokine and chemokine expression after additional stimuli, such as trauma or endotoxemia. Our data indicate that chronic ethanol feeding increased Kupffer cell TNF-*α* release by sensitization to LPS. This may explain the increased susceptibility to infections of trauma victims with a history of chronic ethanol abuse.

## Figures and Tables

**Table 1:** Experimental groups.

	Ethanol-fed (EtOH)	Pair-fed (Ctrl)	Chow-fed (chow)
Mice strain	*cis*-NF-*κ*B^EGFP^	*cis*-NF-*κ*B^EGFP^	C57BL/6
Examination of liver tissue, blood	*n* = 15	*n* = 15	*n* = 15
Soxhlet extraction	*n* = 5	*n* = 5	*n* = 5
Isolation of Kupffer cells	*n* = 15	*n* = 15	*n* = 20

**Table 2:** Primers used for qRT-PCR of Kupffer cells.

Gene name	RefSeq accession no.	UniGene no.
Mouse CXCL-1	NM_008176.2	Mm.21013
Mouse IL-6	NM_031168.1	Mm.1019
Mouse MMP9	NM_013599.2	Mm.4406
Mouse NOS2	NM_010927.3	Mm.2893
Mouse TNF	NM_013693.2	Mm.1293

CXCL-1: chemokine (C-X-C motif) ligand 1; IL-6: interleukin 6; MMP9: matrix metalloproteinase 9; NOS2: nitric oxide synthase 2 (inducible); TNF: tumor necrosis factor.

**Table 3:** CD11b and CD68 expression of F4/80^+^ cells in the liver.

	Ethanol-fed (EtOH)	Pair-fed (Ctrl)
%CD11b^+^ of F4/80^+^ cells	24.4 ± 2.3	12.3 ± 1.2
%CD68^+^ of F4/80^+^ cells	41.5 ± 5.1	12.9 ± 1.8

CD11b and CD68 expression of liver F4/80^+^ cells, isolated from EGFP reporter gene mice after 4 weeks lasting Lieber-DeCarli pair-feeding regime. Data are percentages (mean ± SEM) of five mice in each group with similar results.
